# Tetanus: A disease not to be forgotten

**DOI:** 10.1590/0037-8682-0586-2022

**Published:** 2023-03-06

**Authors:** Ayşe Sağmak Tartar, Ayhan Akbulut, İsmail Demirel

**Affiliations:** 1 Firat University, Faculty of Medicine, Department of Infectious Diseases and Clinical Microbiology, Elazig, Turkey .; 2 Firat University, Faculty of Medicine, Department of Anesthesiology and Reanimation, Elazig, Turkey .

A 62-year-old woman presented to the emergency department of our hospital after falling from a height. A dirty open wound contaminated with soil was observed on the left thumb, which was sutured (Figure 1). A tetanus vaccine was administered but not tetanus immune globulin(TIG). Amoxicillin-clavulanate treatment was initiated. Seven days later, the patient presented to the emergency department again with difficulty swallowing and a sore throat. She was examined by doctors in the neurology, gastroenterology, and otorhinolaryngology departments. However, no pathology was diagnosed. The patient also started experiencing spasms toward the back of the neck. The spatula test for tetanus was positive. Risus sardonicus and trismus were also observed (Figure 2). The patient reported that she had not received a tetanus vaccine in adulthood. The patient was isolated in a dark and quiet room and the wound was debrided regularly by plastic surgeons. Treatment included the administration of meropenem, linezolid, metronidazole, and TIG. The patient experienced cardiac arrest on day 2 of hospitalization and responded to cardiopulmonary resuscitation. She was placed under sedation because of severe muscular contractions, and a tracheostomy was performed. On day 20, the patient’s contractions were controlled. The patient was discharged and advised to complete her vaccinations for tetanus, diphtheria, and pertussis as per the immunization schedule.

**FIGURE: 1:**
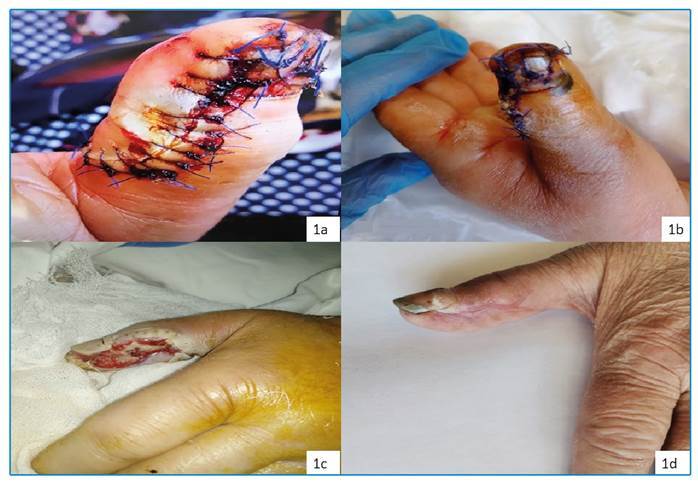
A dirty open wound contaminated with soil on the left thumb. **1a:** the first day; **1b:** the 10th day; **1c:** after debridement; **1d:** the 42nd day.

**FIGURE 2:**
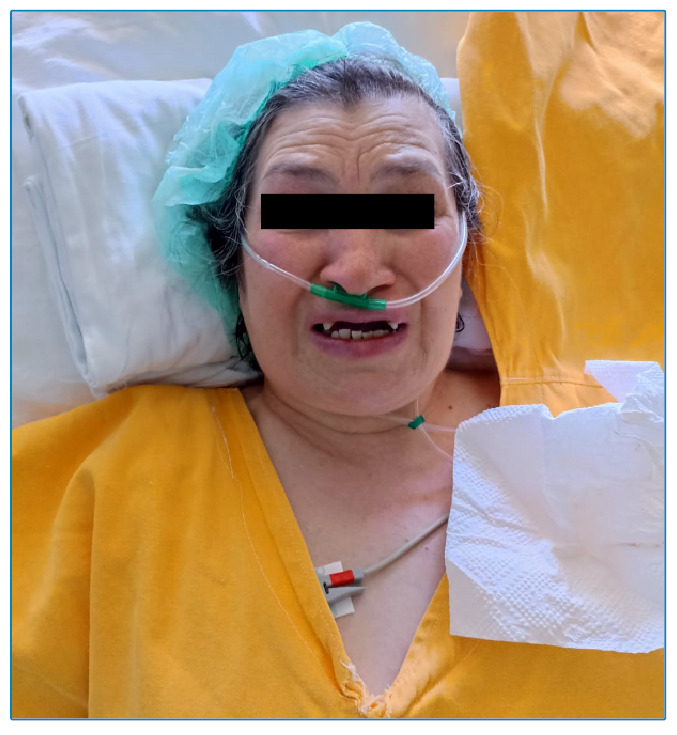
Paitent with observable risus sardonicus and trismus.

Although the incidence of tetanus in the developed world is markedly low, it remains a threat, particularly in countries where vaccination is insufficient^1^. Considering the increasing anti-vaccination trend and insufficient immunization among adults, tetanus is a disease that doctors should familiarize^2^.

## References

[1] Havers FP, Moro PL, Hunter P, Hariri S, Bernstein H (2020). Use of Tetanus Toxoid, Reduced Diphtheria Toxoid, and Acellular Pertussis Vaccines: Updated Recommendations of the Advisory Committee on Immunization Practices - United States, 2019. MMWR Morb Mortal Wkly Rep.

[2] Tosun S, Batirel A, Oluk AI, F Aksoy, E Puca, F Bénézit (2017). Tetanus in adults: results of the multicenter ID-IRI study. Eur J Clin Microbiol Infect Dis.

